# Enterobacteriaceae in soils and atmospheric dust aerosol accumulations of Moscow city

**DOI:** 10.1016/j.crmicr.2022.100124

**Published:** 2022-03-04

**Authors:** Аnna М. Glushakova, Аleksey V. Kachalkin, Tatiana V. Prokof'eva, Ludmila V. Lysak

**Affiliations:** a M.V. Lomonosov Moscow State University, Moscow, 119234, Russian Federation; b I.I. Mechnikov Research Institute of Vaccines and Sera, Moscow, 105064, Russian Federation; c G.K. Skryabin Institute of Biochemistry and Physiology of Microorganisms of RAS, Pushchino, 142290, Russian Federation

**Keywords:** Soil, Dust, Anthropogenic impact, Feces contamination, *Escherichia coli*, *Enterobacteriaceae*, Antibiotic resistance

## Abstract

The topsoils and atmospheric dust aerosols of the various areas of the city of Moscow were studied. Most of the dust samples contained a considerable number of particles enriched in phosphorus - a sign of contamination by feces. A variety of *Enterobacteriaceae* species, including opportunistic and pathogenic species, were isolated from the topsoil and dust samples and identified using 16S rDNA nucleotide sequences: *Enterobacter aerogenes, E. agglomerans, E. cloacae, E. kobei, E. nimipressuralis, Escherichia coli, Citrobacter europaeus, Klebsiella granulomatis, K. grimontii, K. oxytoca, K. quasipneumoniae, K. variicola, Kluyvera ascorbate, Kluyvera intermedia, Leclercia adecarboxylata, Salmonella enterica* and *Trabulsiella guamensis*. The greatest diversity of pathogens was isolated from spring soil and dust samples immediately after spring snowmelt. Antibiotic resistance of the isolated *E. coli* strains was tested using disks with a wide range of antimicrobial drugs: Amoxicillin, Ampicillin, Meropenem, Pefloxacin, Streptomycin, Ticarcillin+clavulanic acid, Fosfomycin, Ceftibuten, Ciprofloxacin. Resistance was observed in more than 22% of *E. coli* strains. The traffic area had a significant number of antibiotic-resistant *E. coli* strains, clearly indicating a high health risk from soil and dust exposure.

## 1 Introduction

Large cities and megacities around the world form urban ecosystems that differ greatly from natural ecosystems in terms of weather, physical and chemical properties of soils, and microbial biodiversity in the atmosphere, soil, water, and plants (Pickett et al., 2001). The formation and development of urban ecosystems is expected to flourish in all regions, albeit at different rates and with different characteristics for each nation (World Urbanization Prospects 2018: Highlights).

Moscow, the capital and largest city of Russia, as well as one of the largest cities in the world, suffers from excessive anthropogenic impact related to a growing population and a huge seasonal tourism industry. On the list of the most populous cities in the world, Moscow ranks 24th (out of 81 cities with more than 5 million inhabitants) (estimate from UN, United Nations).

The impact of urbanization and industrialization affects all natural habitats in the urban environment: plants, waters and soils (Singh, Singh, 2017; Barletta, Lima, 2019). Land cover largely determines the ecological and sanitary status of cities around the world, which vary in size and population (Holcomb et al., 2020; Glushakova et al., 2021). Soils can clean themselves from various polluting solids, liquids and gasses (Andreoni et al., 2004; Braun et al., 2006; Dobrovol'skaya et al., 2015), disinfect the urban environment from opportunistic and pathogenic microorganisms and their toxins (Baumgardner, 2012; Samaddar et al., 2021), and regulate the molecular composition of air in the urban environment (Giltrap et al., 2021). Main components of atmospheric solid aerosols in cities are also soil and sediment particles, and primary biological aerosol particles, i.e. microorganisms (aeroplankton) and their remains (Després et al., 2012; Prokof'eva et al., 2017). Urban soils and dust aerosols may contain not only saprotrophic microorganisms, but also opportunistic and pathogenic species of bacteria, yeasts and mycelial fungi, with which humans can easily come into contact (Prokof'eva et al., 2021). It is especially unsafe for immunosuppressed people and children whose immune system is not fully developed. Therefore, urban microbes and soil dust aerosols form a common system in cities and megacities, and the characteristics of their microbial diversity should be studied simultaneously.

We devoted our research to the evaluation of the diversity of *Enterobacteriaceae* in the topsoils and dust aerosols from different areas of the city of Moscow. The specific objective was to verify dust possible contamination by feces and to evaluate the resistance of *E. coli* strains to antimicrobial drugs.

## 2 Materials and methods

### 2.1 Areas of investigation and sampling

Sampling was carried out in different urban sites: recreational areas which are located near residential and public places, and areas with intensive traffic. The locations and their characteristics are listed in Table 1.

**Table 1:** Locations and their characteristics.

Locations	GPS coordinates	Season	Year	Kind of sample	Time of accumulation (for dust aerosols)	Code
Recreational area						
Botanical Garden of the Lomonosov Moscow State University	55.7049, 37.5270	Summer	2019	Dust Soil	summertime	D2 S2
Leo Tolstoy House-Museum in Khamovniki	55.7342, 37.5862	Summer	2019	Dust Soil	summertime	D1 S1
Intensive traffic area						
Lane tunnel at the crossing of the Third Transport Ring and the Kutuzovsky Avenue - the surface of a 40 cm-high asphalt-concrete block	55.7383, 37.5373	Autumn Spring	2020 2021	Dust Dust Soil	Summertime wintertime	D3 D5 S5
Lane tunnel at the crossing of the Third Transport Ring and the Kutuzovsky Avenue, the road-facing side of a noise-protection screen at heights of 150–200 cm.	55.7382, 37.5377- 55.7389, 37.5370	Autumn Spring	2020 2021	Dust Dust Soil	Summertime wintertime	D4 D6 S6
Both sides of 70 cm-high metal barriers along the road on a 20 m-high bridge above the railway (Kievsky overpass)	55.7365, 37.5397	Spring	2021	Dust	wintertime	D7

Dust material for the study was collected by direct settling of aerosol particles from the atmosphere in open containers at the recreational areas on summertime. We used an approved method for collecting dry atmospheric dust aerosol, similar methods have been previously tested and described by other researchers for different areas (Tentyukov, 2008, 2013; Prokof'eva et al., 2021). Atmospheric aerosol fell into open plastic containers during August–September. Samples from asphalt-concrete block, metal road barriers and anti-noise screens were swept away in the traffic area. Twice a year, all roadside structures are washed in Moscow. We collected samples before cleaning - in September (accumulated in summer) and in early March (accumulated in winter).

Soil samples were collected from the topsoil using the standard method (Glushakova et al., 2021). A total of 4 topsoil samples (10–15 g each) and 7 dust samples (10 g each) were collected.

This work can be considered as the first simultaneous study of urban soils and dust aerosols in the Moscow region. Of course, the number of samples we have studied so far is very limited and does not allow us to draw global conclusions. The study of urban soils and dust in the Moscow region will be continued in the near future.

### 2.2 Morphological analyzes

Morphological composition of seven dust samples was examined with a scanning electron microscope 6610 LV (JEOL) with an energy-dispersive spectrometer INCA X-ACT (Oxford Instruments). The bulk elemental composition was estimated in native samples during microscopy by energy-dispersive X-ray spectrometer (for the whole sample as well as for individual aggregates). Each of the 7 dust samples were attached to a double-sided cloth tape, spraying was carried out with silver. The samples were examined at different magnifications. To search for fecal microfragments, the composition of aggregates ranging in size from several hundred to 5–10 µm consisting of decomposed organic residues that have lost their original cellular structure was checked. Hyphae of fungi served as an additional indication of a possible origin from feces. The phosphorus content in microfragments was determined using an energy dispersive microanalyzer.

### 2.3 Microbiological analyzes and species identification

The abundance and taxonomic structure of *Enterobacteriaceae* were studied using a surface plating method on solid media. From each sample, 3 sub-samples (1 g each) were collected after thorough mixing and analyzed. Soil and dust samples were collected and poured with sterile water to obtain a dilution of 1:1000. To desorb the bacteria from the surface of soil and dust particles, water suspensions were treated by ultrasound in the device UDZN (22 kHz, 0.44A, 2 min). Thus, 33 prepared suspensions were plated in six replicates each on REBECCA® EB (bioMerieux, Inc., France), a highly selective chromogenic medium (NF Validation EN ISO 16,140). A total of 198 plates were incubated at 25 °C for 5–7 days. The grown bacterial colonies were classified into morphological types under a dissecting microscope and the number of colonies of each type was counted. From each morphotype, 10 – 15 colonies were isolated into a pure culture. Identification was based on 16S rDNA nucleotide sequence data using the BLAST NCBI. DNA isolation from pure bacterial cultures was performed using the PrepMan Ultra Sample Preparation Reagent (Applied Biosystems, USA) kit according to the manufacturer's recommendations. Sequencing of the PCR product of the variable sequence v3-v4 of the 16S rRNA region was performed according to the standard protocol of the MicroSeq 500 16S rDNA Bacterial Identification Kit (MicroSEQ™ 500 16S rDNA Identification User Guide, Thermo Fisher, USA) using standard fD1/rD1 primers (Baker et al., 2003; Manucharova et al., 2008). DNA sequencing was performed using an ABI Prism 3130 genetic analyzer. To analyze the obtained electrophoretograms and nucleotide sequences, we used MicroSeq ID v.2.0 (Applied Biosystems, USA) software and the validated MicroSeq ID 16S rDNA 500 Library v2.0. Analysis of 16S rDNA sequences was performed at Syntol (Moscow, Russia).

### 2.4 Antibiotic sensitivity of *E. coli* strains

Antibiotic susceptibility of *E. coli* strains was tested using Mueller-Hinton agar (HiMedia Laboratories Pvt. Ltd., India) which is a standard medium for disk diffusion method (Bauer-Kirby test) as per the guidelines of Global Laboratory Standards for a Healthier World (CLSI) of USA. Disks were tested with a wide range of antimicrobial drugs (HiMedia Laboratories Pvt. Ltd., India): Amoxicillin 10 (µg/disk) (AMO), Ampicillin 10 (µg/disk) (AMP), Meropenem 10 (µg/disk) (MER), Pefloxacin 5 (µg/disk) (PEF), Streptomycin 300 (µg/disk) (STR), Ticarcillin+clavulanic acid 75 (µg/disk) (TIC), Fosfomycin 200 (µg/disk) (FOS), Ceftibuten 30 (µg/disk) (CEF), Ciprofloxacin 10 (µg/disk) (CIP). As a control, the reference strain of *E. coli* ATCC 2592 was used, which is recommended by the CLSI (https://clsi.org). A total of 205 strains of *E. coli* were tested: 118 strains were isolated from dust samples (60 strains were isolated from the intensive traffic area and 58 from the recreational area) and 87 strains were isolated from soil samples (44 strains were isolated from the intensive traffic area and 43 - from the recreational area). Each of the 205 strains was tested in three plate replicates for each of the 9 antimicrobial drugs.

### 2.5 Data analyses

The relative abundances of species were calculated as share (%) of colonies that appeared on the plates. Statistical data processing and graphical presentation of the obtained results were carried out using Excel 2010 (Microsoft, USA) and Statistica 8.0 (StatSoft, USA) programs. The Fisher's test was used for comparing average data on the bacteria abundance after determining normality of their distribution by the Shapiro-Wilk test. Cluster analysis (nearest neighbor method, Euclidean distance measure) was applied for comparing the species structures based on the relative abundance of bacteria species.

## 3 Results

### 3.1 Fecal contamination of dust aerosols

**Fig. 1:**
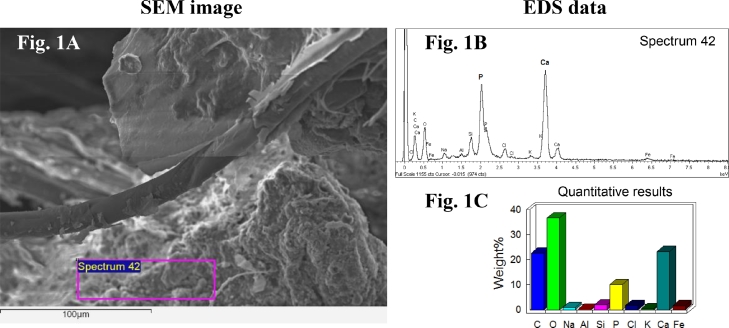
A representative data of SEM-EDS analysis (scanning electron microscopy (Fig. 1A) and energy dispersive X-ray spectroscopy (Fig. 1B) with elemental microanalysis (Fig. 1C)) of microparticles with high concentrations of carbon (C), oxygen (O), phosphorus (P) and calcium (Ca) in dust aerosol sample.

Using a JEOL JSM-6610LV scanning electron microscope with an INCAx-act energy dispersive detector seven dust samples were examined (Fig. 1A). Dried fragments of feces were found in all dust samples. They were identified by a high content of phosphorus (Fig. 1B, 1C). Dust microfragments with a concentration of phosphorus more than 50 g/kg were found in each dust sample.

### 3.2 Diversity of *Enterobacteriaceae*

All representatives of *Enterobacteriaceae* isolated from soils and atmospheric dust aerosols were classified into 8 genera and 17 species as follows: *Citrobacter europaeus*, 5 *Enterobacter* spp, *E. coli*, 5 *Klebsiella* spp, 2 *Kluyvera* spp, *Leclercia adecarboxylata, Salmonella enterica, Trabulsiella guamensis* (Table 2).

**Table 2:** Relative abundances (%) * with standard deviations of *Enterobacteriaceae* isolated from the dust and soil samples.

Samples/Species	D-1	D-2	D-3	D-4	D-5	D-6	D-7	S-1	S-2	S-5	S-6
*Citrobacter europaeus*	– **	–	–	–	8.23 ± 0.01	6,01 ± 0.03	–	–	13.98 ± 0.60	10.56 ± 0.05	9.17 ± 0.11
*Enterobacter aerogenes*	–	–	–	–	3.24 ± 0.01	4.12 ± 0.01	–	–	2.89 ± 0.07	1.07 ± 0.05	2.71 ± 0.02
*Enterobacter agglomerans*	–	–	45.06 ± 0.01	57.39 ± 0.02	4.11 ± 0.01	3.28 ± 0.03	4.51 ± 0.01	–	–	16.76 ± 0.08	1.16 ± 0.04
*Enterobacter cloacae*	–	–	–	–	2.08 ± 0.01	3.07 ± 0.01	–	–	–	9.25 ± 0.08	4.22 ±0 .04
*Enterobacter kobei*	–	–	–	–	3.66 ± 0.02	3.56 ± 0.01	3.89 ± 0.03	–	–	0.12 ± 0.02	2.09 ±0 .03
*Enterobacter nimipressuralis*	–	–	30.73 ± 0.01	21.0 ± 0 .04	4.81 ± 0.01	2.82 ± 0.01	8.04 ±0 .09	–	–	1.34 ± 0.06	1.56 ± 0.05
*E. coli*	89.12 ± 0.01	100	24.21 ± 0.01	21.53 ± 0.02	17.80 ± 0.01	19.88 ± 0.01	15.74 ± 0.01	67.24 ± 0.65	83.13 ± 0.54	20.71 ± 0.02	22.09 ± 0.02
*Klebsiella granulomatis*	–	–	–	–	11.19 ± 0.01	10.98 ± 0.01	26.87 ± 0.02	–	–	7.67 ± 0.03	11.47 ± 0.05
*Klebsiella grimontii*	–	–	–	–	10.31 ± 0.01	11.27 ± 0.01	–	4.08 ± 0.04	–	3.14 ± 0.10	7.02 ± 0.16
*Klebsiella oxytoca*	10.88 ± 0.01	–	–	–	11.05 ± 0.01	12.05 ± 0.02	–	2.17 ± 0.05	–	5.89 ± 0.04	8.70 ± 0.02
*Klebsiella quasipneumoniae*	–	–	–	–	11.07 ± 0.01	10.19 ± 0.01	40.95 ± 0.05	–	–	2.43 ± 0.02	5.04 ± 0.12
*Klebsiella variicola*	–	–	–	–	12.45 ± 0.03	10.21 ± 0.01	–		–	13.68 ± 0.11	10.91 ± 0.01
*Kluyvera ascorbata*	–	–	–	–	–	–	–	14.86 ± 0.38	–	0.92 ± 0.07	0.94 ± 0.05
*Kluyvera intermedia*	–	–	–	–	–	–	–		–	3.67 ± 0.04	2.21 ± 0.01
*Leclercia adecarboxylata*	–	–	–	–	–	–	–	–	–	–	9.04 ± 0.04
*Salmonella enterica*	–	–	–	–	–	2.56 ± 0.01	–	–	–	–	–
*Trabulsiella guamensis*	–	–	–	–	–	–	–	11.65 ± 0.29	–	2.79 ± 0.20	1.67 ± 0.32

*A species average percentage of the total number of bacteria in a sample.

**not found.

A highest diversity of *Enterobacteriaceae* spp*.* was observed in spring soil and dust samples from the intensive traffic area. In contrast, dust and soil samples from the recreational area had the lowest diversity of *Enterobacteriaceae* during summertime (Table 2).

The two-way ANOVA showed that the species number of *Enterobacteriaceae* most significantly depended on the season of sampling (*F* = 49.45, *p* < 0.01), and to a lesser extent on the matrix type (*F* = 6.46, *p* < 0.05).

**Fig. 2:**
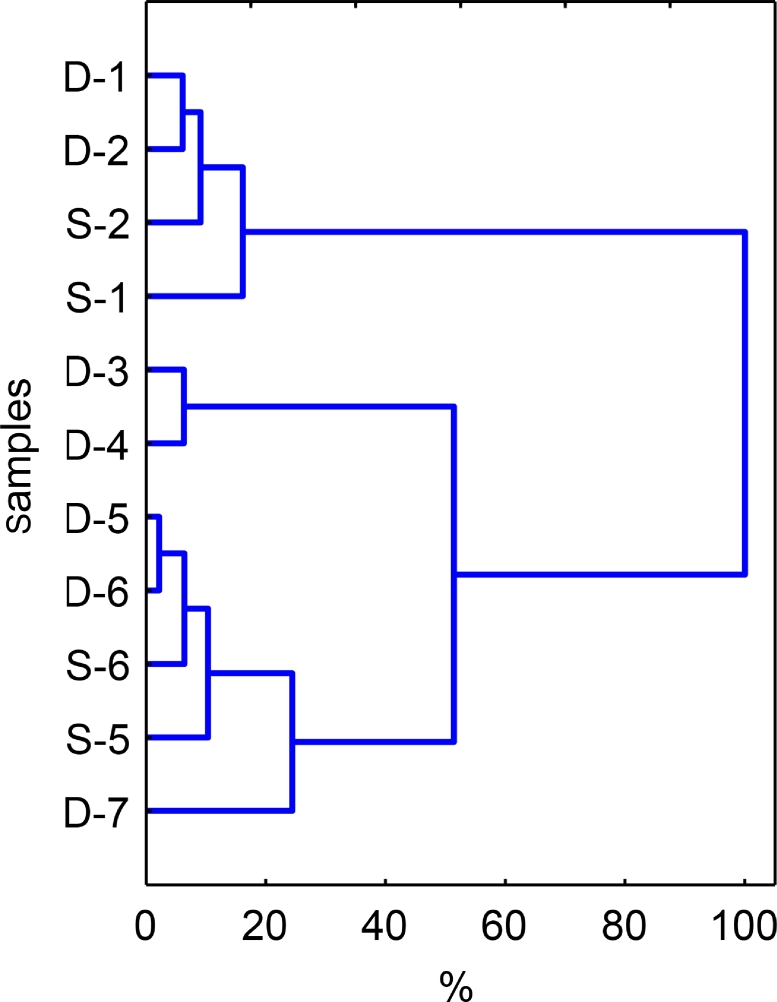
Dendrogram generated using the nearest neighbor method and Euclidean distances, showing similarities (%) of average relative abundance values of bacterial communities in the studied samples (see Table 2).

Clusterization of the studied soils and dust aerosols using the data on the relative abundance of *Enterobacteriaceae* species demonstrated that the season and the type of the studied area affect the structure of bacteria communities (Fig. 2).

*E. coli* was isolated from all soil and dust samples analyzed at varying relative abundance (Table 2). *S. enterica* was isolated only from the dust aerosol sample from the intensive traffic area (wintertime accumulation) (Table 2).

### 3.3 *E. coli* strains with antibiotic resistance

A total of 46 out of 205 strains were resistant to one or more antibiotic (Table 3). Of these, only 16 strains of *E. coli* were isolated from dust and soil samples originating from the recreational area, and the remaining 30 strains were isolated from dust and soil samples from the intensive traffic area.

**Table 3:** Strains of *Escherichia coli* isolated from dust and soil of Moscow with antibiotic susceptibility (retarded growth with standard deviations, mm) below the reference values**.

Antibiotics/Strains	AMO***	AMP	MER	PEF	STR	TIC	FOS	CEF	CIP
D1–1	16.7 ± 0.03	14.3 ± 0.07	16.7 ± 0.07	16.7 ± 0.27	20.3 ± 0.20	11.3 ± 0.43	26.3 ± 0.09	11.7 ± 0.15	16.3 ± 0.07
D1–7	17.7 ± 0.03	14.0 ± 0.03	16.7 ± 0.09	17.7 ± 0.07	20.7 ± 0.03	10.7 ± 0.21	23.0 ± 0.07	12.3 ± 0.15	16.3 ± 0.12
D1–8	21.0 ± 0.44	14.0 ± 0.12	17.0 ± 0.06	15.7 ± 0.09	6.0 ± 0.07	8.0 ± 0.15	18.3 ± 0.15	10.3 ± 0.06	20.7 ± 0.17
D1–12	14.8 ± 0.06	19.0 ± 0.12	14.7 ± 0.03	16.3 ± 0.09	6.7 ± 0.12	7.0 ± 0.07	27.7 ± 0.07	8.7 ± 0.09	19.3 ± 0.06
D1–19	16.0 ± 0.06	16.0 ± 0.06	16.3 ± 0.03	16.3 ± 0.03	6.0 ± 0.20	8.0 ± 0.13	22.7 ± 0.07	11.7 ± 0.27	19.3 ± 0.10
D2–3	9.0 ± 0.03	14.0 ± 0.03	9.7 ± 0.07	16.7 ± 0.15	5.7 ± 0.07	6.3 ± 0.15	27.3 ± 0.10	7.5 ± 0.18	20.0 ± 0.12
D2–15	16.0 ± 0.09	14.0 ± 0.12	18.0 ± 0.07	16.3 ± 0.06	8.3 ± 0.06	10.0 ± 0.34	19.7 ± 0.07	9.7 ± 0.15	16.7 ± 0.06
D3–4	15.0 ± 0.03	17.3 ± 0.09	16.3 ± 0.03	15.4 ± 0.15	6.7 ± 0.15	25.3 ± 0.15	25.0 ± 0.07	20.0 ± 0.03	21.7 ± 0.09
D3–7	14.0 ± 0.09	14.0 ± 0.12	15.7 ± 0.03	17.0 ± 0.12	8.3 ± 0.03	18.3 ± 0.09	21.0 ± 0.03	10.3 ± 0.15	22.3 ± 0.10
D3–8	14.0 ± 0.03	15.3 ± 0.06	16.3 ± 0.09	18.0 ± 0.15	9.7 ± 0.09	20.7 ± 0.09	16.7 ± 0.07	11.3 ± 0.12	21.7 ± 0.09
D3–9	14.7 ± 0.07	14.0 ± 0.17	16.7 ± 0.15	17.0 ± 0.12	14.3 ± 0.07	10.3 ± 0.27	20.3 ± 0.06	18.3 ± 0.09	22.7 ± 0.09
D3–10	16.0 ± 0.12	16.3 ± 0.06	13.3 ± 0.03	7.3 ± 0.26	23.3 ± 0.12	23.3 ± 0.07	21.7 ± 0.07	20.3 ± 0.06	25.3 ± 0.17
D3–13	17.0 ± 0.06	16.0 ± 0.09	13.3 ± 0.06	20.0 ± 0.38	24.0 ± 0.07	25.7 ± 0.03	25.7 ± 0.09	19.3 ± 0.09	20.0 ± 0.09
D3–15	14,0 ± 0,06	14.0 ± 0.07	15.3 ± 0.03	15.0 ± 0.12	6.0 ± 0.07	6.0 ± 0.13	26.7 ± 0.03	19.0 ± 0.07	22.7 ± 0.18
D3–22	16.0 ± 0.03	16.7 ± 0.03	14.7 ± 0.07	17.0 ± 0.12	18.3 ± 0.15	19.3 ± 0.09	21.0 ± 0.10	19.3 ± 0.03	23.3 ± 0.09
D3–32	16.7 ± 0.07	14.0 ± 0.03	15.3 ± 0.03	18.7 ± 0.07	10.0 ± 0.13	15.0 ± 0.07	20.3 ± 0.07	19.0 ± 0,07	17.3 ± 0.15
D4–1	16.0 ± 0.06	15.7 ± 0.06	16.0 ± 0.07	12.7 ± 0.09	16.3 ± 0.06	9.7 ± 0.09	20.3 ± 0.03	9.3 ± 0.06	21.0 ± 0.10
D4–19	13.9 ± 0.03	15.0 ± 0.07	17.3 ± 0.07	18.0 ± 0.18	18.3 ± 0.03	23.7 ± 0.03	16.0 ± 0.07	22.0 ± 0.07	25.3 ± 0.12
D5–4	22.0 ± 0.03	16.3 ± 0.09	15.3 ± 0,09	17.0 ± 0.07	11.0 ± 0.12	21.7 ± 0.10	23.7 ± 0.06	20.3 ± 0.18	22.3 ± 0.09
D5–12	17.1 ± 0.03	16.0 ± 0.06	14.2 ± 0,06	20.2 ± 0.12	14.2 ± 0.07	23.4 ± 0.12	12.6 ± 0.06	20.5 ± 0.13	20.4 ± 0.07
D5–28	22.0 ± 0.09	18.0 ± 0.12	16.0 ± 0,07	20.7 ± 0.20	14.3 ± 0.15	21.9 ± 0.09	22.7 ± 0.03	21.3 ± 0.06	16.3 ± 0.09
D5–46	19.0 ± 0.06	12.0 ± 0.20	17.0 ± 0.09	20.7 ± 0.12	13.7 ± 0.15	20.7 ± 0.03	22.3 ± 0.06	18.7 ± 0.09	21.3 ± 0.03
D5–56	11.0 ± 0.18	16.3 ± 0.09	15.3 ± 0.03	12.0 ± 0.23	17.3 ± 0.07	16.0 ± 0.12	27.7 ± 0.07	13.7 ± 0.09	23.0 ± 0.07
D6–8	13.9 ± 0.03	16.3 ± 0.15	16.1 ± 0.03	21.4 ± 0.17	12.8 ± 0.07	22.8 ± 0.07	22.8 ± 0.07	19.2 ± 0.10	18.2 ± 0.07
D6–9	12.3 ± 0.07	19.0 ± 0.15	14.3 ± 0.07	13.3 ± 0.06	16.3 ± 0.10	21.7 ± 0.09	24.0 ± 0.07	11.7 ± 0.15	23.0 ± 0.15
D6–11	17.0 ± 0.09	14.0 ± 0.06	14.2 ± 0.06	17.6 ± 0.15	12.2 ± 0.07	21.0 ± 0.07	22.8 ± 0.09	16.4 ± 0.09	21.8 ± 0.06
D6–14	14.8 ± 0.12	16.5 ± 0.03	14.0 ± 0.06	17.6 ± 0.09	12.2 ± 0.09	22.2 ± 0.03	21.4 ± 0.03	17.9 ± 0.19	22.4 ± 0.13
D6–23	17.3 ± 0.12	19.0 ± 0.12	14.7 ± 0.03	18.0 ± 0.13	15.7 ± 0.09	22.7 ± 0.06	21.7 ± 0.06	11.3 ± 0.15	22.7 ± 0.09
D6–24	19.0 ± 0.09	14.0 ± 0.00	15.7 ± 0.03	15.7 ± 0.06	12.3 ± 0.07	20.7 ± 0.03	21.3 ± 0.03	9.7 ± 0.12	21.7 ± 0.17
D6–26	13.6 ± 0.07	15.4 ± 0.09	15.2 ± 0.07	14.2 ± 0.06	14.0 ± 0.03	23.0 ± 0.07	24.8 ± 0.09	18.0 ± 0.12	14,5 ± 0,12
D7–11	18.7 ± 0.09	14.0 ± 0.18	15.3 ± 0.03	16.7 ± 0.09	12.3 ± 0.07	21.9 ± 0.06	20.3 ± 0.09	20.3 ± 0.09	20.3 ± 0.06
D7–21	16.0 ± 0.03	14.7 ± 0.06	15.6 ± 0.03	20.5 ± 0.03	14.5 ± 0.13	20.5 ± 0.03	20.8 ± 0.06	16.8 ± 0.12	19.2 ± 0.06
S1–8	13.8 ± 0.03	16.3 ± 0.06	16.4 ± 0.03	18.8 ± 0.09	14.4 ± 0.03	21.8 ± 0.10	24.7 ± 0.07	19.8 ± 0.10	20.1 ± 0.13
S1–17	16.1 ± 0.07	17.4 ± 0.03	16.2 ± 0.07	15.5 ± 0.09	14.8 ± 0.03	18.6 ± 0.07	16.1 ± 0.03	18.0 ± 0.03	15.8 ± 0,10
S2–21	16.8 ± 0.03	14.7 ± 0.07	17.4 ± 0.07	19.2 ± 0.23	12.1 ± 0.03	17.5 ± 0.15	14.0 ± 0.15	20.1 ± 0.09	20.8 ± 0.13
S5–2	13.8 ± 0.12	16.5 ± 0.07	15.7 ± 0.03	22.4 ± 0.12	12.2 ± 0.07	22.6 ± 0.12	16.3 ± 0.03	21.4 ± 0.03	16.1 ± 0.03
S5–16	14.2 ± 0.03	16.2 ± 0.15	16.1 ± 0.03	15.8 ± 0.24	12.4 ± 0.20	18.2 ± 0.23	15.2 ± 0.12	22.5 ± 0.15	16.8 ± 0.03
S5–27	15.1 ± 0.09	15.8 ± 0.25	16.0 ± 0.07	17.4 ± 0.03	12.2 ± 0.18	17.5 ± 0.03	16.8 ± 0.03	21.8 ± 0.07	17.9 ± 0.06
S6–11	14.6 ± 0.15	16.1 ± 0.06	15.9 ± 0.07	17.8 ± 0.18	12.8 ± 0.07	21.4 ± 0.03	16.4 ± 0.03	21.6 ± 0.03	20.1 ± 0.03
S6–14	17.3 ± 0.03	16.2 ± 0.06	16.2 ± 0.12	17.4 ± 0.13	14.2 ± 0.10	14.2 ± 0.07	15.8 ± 0.03	20.9 ± 0.09	18.4 ± 0.07
S6–31	15.1 ± 0.06	15.8 ± 0.07	12.7 ± 0.07	18.2 ± 0.12	14.0 ± 0.07	22.5 ± 0.03	12.6 ± 0.07	17.9 ± 0.09	16.5 ± 0.15
S6–38	15.2 ± 0.03	16.1 ± 0.06	16.6 ± 0.03	17.1 ± 0.12	14.0 ± 0.09	21.8 ± 0.09	16.5 ± 0.03	22.5 ± 0.12	18.2 ± 0.03
S6–67	16.8 ± 0.07	16.0 ± 0.06	16.2 ± 0.07	18.8 ± 0.12	12.6 ± 0.07	19.1 ± 0.06	16.1 ± 0.03	22.9 ± 0.06	19.2 ± 0.07
S6–74	17.0 ± 0.03	16.7 ± 0.07	16.4 ± 0.21	17.5 ± 0.03	14.2 ± 0.10	19.0 ± 0.07	15.8 ± 0.09	21.4 ± 0.06	22.5 ± 0.03
S6–77	17.1 ± 0.12	17.4 ± 0.06	13.5 ± 0.09	17.4 ± 0.06	13.8 ± 0.12	22.0 ± 0.73	17.4 ± 0.13	22.8 ± 0.07	21.4 ± 0.13
S6–81	13.9 ± 0.03	14.2 ± 0.07	15.9 ± 0.06	18.2 ± 0.06	14.2 ± 0.03	22.8 ± 0.03	12.9 ± 0.13	20.1 ± 0.03	20.2 ± 0.06
Reference values**	**14–17**	**14–16**	**14–15**	**16–21**	**12–14**	**22–23**	**13–15**	**18–20**	**16–20**

*– Values indicating antibiotic resistance are highlighted in gray.

** – Values for the control strain ATCC 2592.

***AMO - Amoxicillin; AMP - Ampicillin; MER - Meropenem; PEF - Pefloxacin; STR - Streptomycin; TIC - Ticarcillin+clavalanic acid; FOS - Fosfomycin; CEF - Ceftibuten; CIP - Ciprofloxacin.

## 4 Discussion

### 4.1 High phosphorus concentration in dust aerosols microfragments

Based on the P (phosphorus) concentrations, the dust microfragments can originate from P-enriched soils. However, as it has already been shown, the main source of P enrichment of Moscow soils is the feces of domestic animals (Stroganova et al., 1998). The search and identification of morphologically recognizable microfragments of fecal matter in dust aerosols is very controversial. We relied on the presence of a significant concentration of phosphorus (P). In cases where the concentration was higher than it usually in soils, we identified the analyzed microfragments as fecal matter. It is known that soils have a phosphorus concentration between 0.2–5 g/kg (Theory and Practice of chemical analysis of soil, 2006). On the other hand, dogs have a high protein diet and their feces are known to have very high P concentrations. Based on the results of our recent study, the microscopized dog feces that had been lying on the soil surface for several weeks (during the summer dry period) and partially destroyed showed an extremely high concentration of P - up to 130 g/kg (Prokof'eva et al., 2021).

### 4.2 Isolated *Enterobacteriaceae* species

The high relative abundance of the sanitary indicator *E. coli* in the microbial communities of soil and dust in the city of Moscow is an important indication of anthropogenic impact on soil - the presence of dangerous fecal contamination (Hardina, Fujioca, 1991; Campos Pinilla et al., 2015).

In the studied soil and dust aerosol samples of the city of Moscow, the genus *Klebsiella* was represented by five species, with the highest diversity in the samples from the intensive traffic area. These species are regularly isolated from clinical material and are involved in the development of opportunistic infections (Raro et al., 2019; Arabaghian et al., 2019; Araujo et al., 2021). Likewise, *Enterobacter* species can cause opportunistic infections in human and animals (Davin-Regli, Pagès, 2015; Zhou et al., 2017; Büyükcam et al., 2018; Annavajhala et al., 2019; Fuentes-Castillo et al., 2021). *Citrobacter europaeus*, isolated in this study from soil and dust samples of the intensive traffic area; was first isolated from the feces of a diarrheal patient and described in 2017 (Ribeiro et al., 2017). *Kluyvera ascorbata* and *Kluyvera intermedia* are potential pathogens and can cause various infections (Carter et al., 2005; Isozaki et al., 2010; Thele et al., 2017; Wang et al., 2018). In our study, they were isolated from soil samples of the intensive traffic area and the recreation area. *Leclercia adecarboxylata* is most commonly isolated as a monomicrobial infection in immunocompromised patients and as part of a polymicrobial infection in immunocompetent patients. It has been described as an emerging human pathogen that can cause severe infections in immunocompromised patients (Spiegelhauer et al., 2019). In our study, this pathogen was isolated from the soil of the intensive traffic area.

The highest diversity of potential pathogens of *Enterobacteriaceae* was isolated from wintertime dust aerosol and soil accumulation immediately after snowmelt.

In winter, under the conditions of a temperate climate with a clear seasonality, a stable snow covers soil for 3 – 5 months. During this period, soils under complex anthropogenic impact do not fulfill their ecological function as a "bacterial filter" or fulfill it insufficiently (Lysak, Lapygina, 2018; Glushakova et al., 2021), which is reflected in the high relative abundance of opportunistic and pathogenic species.

### 4.3 Comparison of antibiotic susceptibility of *E. coli* strains

In 2017, the WHO published the list of microorganisms with the highest antibiotic resistance for which new antibiotics are urgently needed. Representatives of *Enterobacteriaceae* occupy the top positions (www.who.int/news/item/27-02-2017-who-publishes-list-of-bacteria-for-which-new-antibiotics-are-urgently-needed). We observed antibiotic resistance in 22. 4% of *E. coli* strains isolated from urban soils and dust aerosols, with the highest resistance to ticarcillin-clavulanate - an antibiotic from the penicillin group in combination with clavulanic acid (*β*-lactamase inhibitor). We suspect that this is due to the recent over-prescription of this beta-lactam antibiotic to patients in the Moscow region. Most of the antibiotic-resistant *E. coli* strains were from spring soil and dust samples of the intensive traffic area.

Indeed, *E. coli* persistence is likely to be triggered by anthropogenic impact, in analogy with previously reported trends in *Mycobacteria, Shigella, Salmonella* and other pathogenic microorganisms (Savilov et al., 2020). The same is true for strains of the other groups of microorganisms. Similarly, it was recently shown that a high proportion of pathogenic and opportunistic yeast strains of *Candida albicans, C. glabrata, C. krusei, C. lusitaniae, C. parapsilosis*, and *C. tropicalis* isolated from urban soils in Moscow were resistant to one or more widely used antimycotics (Glushakova et al., 2017; Akhapkina et al., 2020).

## 5 Conclusion

The composition of *Enterobacteriaceae* species isolated from dust and soil samples was similar, indicating their close ecological relationship. In the present study, most potentially pathogenic *Enterobacteriaceae* species were isolated from spring soil and dust samples of the intensive traffic area.

Atmospheric dust aerosols, in association with animal feces, act as carriers of microbiological contaminants in urban areas. Fecal contaminants were found in all the areas studied.

Antibiotic resistance was found in more than 22% of *E. coli* strains. A wide range of antimicrobial drugs were tested; however, the highest resistance was found for ticarcilin-clavulanate. The traffic area had a significant number of antibiotic resistant *E. coli* strains, which clearly indicates a high health risk due to the exposure of soil and dust.

The obtained data also allow us to make a preliminary suggestion to avoid the use of beta-lactam antibiotics in the Moscow region at this stage. Local screening with prospective testing of dust aerosols in urban environments would be a valuable tool for ecological and surveillance assessments. Our study also provides a basis for recommendations on dog feces disposal. To date, strict administrative control of pet owners is lacking in Moscow. We plan further metagenomic studies of microbial biodiversity in urban soils and dust aerosols in the Moscow region.

## Funding

This research was carried out as part of the Scientific Project of the State Order of the Government of Russian Federation to Lomonosov Moscow State University No. 121,040,800,174–6 (study of the microbiome of soil and dust: microbial diversity analysis); by Russian Foundation for Basic Research (RFBR), project no. 19–05–50,093 (selection of research sites, sampling, microscopic studies); by Development program of the Interdisciplinary Scientific and Educational School of M.V. Lomonosov Moscow State University «Future Planet and Global Environmental Change» (study of strains resistance).

## CRediT authorship contribution statement

**Аnna М. Glushakova:** Conceptualization, Data curation, Formal analysis, Methodology, Visualization, Writing – original draft. **Аleksey V. Kachalkin:** Conceptualization, Data curation, Visualization, Writing – original draft. **Tatiana V. Prokof'eva:** Funding acquisition, Resources, Data curation, Methodology, Visualization. **Ludmila V. Lysak:** Conceptualization.

## Declaration of Competing Interest

The authors declare that they have no known competing financial interests or personal relationships that could have appeared to influence the work reported in this paper.
